# Reliability of IMU-Derived Static Balance Parameters in Neurological Diseases

**DOI:** 10.3390/ijerph18073644

**Published:** 2021-03-31

**Authors:** Clint Hansen, Maximilian Beckbauer, Robbin Romijnders, Elke Warmerdam, Julius Welzel, Johanna Geritz, Kirsten Emmert, Walter Maetzler

**Affiliations:** 1 Department of Neurology, University Hospital Schleswig-Holstein, Arnold-Heller-Strasse 3, Haus D, 24105 Kiel, Germany; Maximilian.Beckbauer@uksh.de (M.B.); r.romijnders@neurologie.uni-kiel.de (R.R.); e.warmerdam@neurologie.uni-kiel.de (E.W.); j.welzel@neurologie.uni-kiel.de (J.W.); j.geritz@neurologie.uni-kiel.de (J.G.); k.emmert@neurologie.uni-kiel.de (K.E.); w.maetzler@neurologie.uni-kiel.de (W.M.); 2 Digital Signal Processing and System Theory, Institute of Electrical and Information Engineering, Kiel University, Kaiserstrasse 2, 24143 Kiel, Germany

**Keywords:** balance, wearable sensors, reliability, neurology, inertial measurement units

## Abstract

Static balance is a commonly used health measure in clinical practice. Usually, static balance parameters are assessed via force plates or, more recently, with inertial measurement units (IMUs). Multiple parameters have been developed over the years to compare patient groups and understand changes over time. However, the day-to-day variability of these parameters using IMUs has not yet been tested in a neurogeriatric cohort. The aim of the study was to examine day-to-day variability of static balance parameters of five experimental conditions in a cohort of neurogeriatric patients using data extracted from a lower back-worn IMU. A group of 41 neurogeriatric participants (age: 78 ± 5 years) underwent static balance assessment on two occasions 12–24 h apart. Participants performed a side-by-side stance, a semi-tandem stance, a tandem stance on hard ground with eyes open, and a semi-tandem assessment on a soft surface with eyes open and closed for 30 s each. The intra-class correlation coefficient (two-way random, average of the k raters’ measurements, ICC2, k) and minimal detectable change at a 95% confidence level (MDC95%) were calculated for the sway area, velocity, acceleration, jerk, and frequency. Velocity, acceleration, and jerk were calculated in both anterior-posterior (AP) and medio-lateral (ML) directions. Nine to 41 participants could successfully perform the respective balance tasks. Considering all conditions, acceleration-related parameters in the AP and ML directions gave the highest ICC results. The MDC95% values for all parameters ranged from 39% to 220%, with frequency being the most consistent with values of 39–57%, followed by acceleration in the ML (43–55%) and AP direction (54–77%). The present results show moderate to poor ICC and MDC values for IMU-based static balance assessment in neurogeriatric patients. This suggests a limited reliability of these tasks and parameters, which should induce a careful selection of potential clinically relevant parameters.

## 1. Introduction

Maintaining balance seems to be a simple task, but it is an essential prerequisite to stand, walk, and engage in everyday activities [1,2]. The underlying processes to sustain balance are based on the interconnection of the vestibular, visual, and somatosensory systems in the central nervous system [3]. Unconsciously, humans stabilize their gaze and perceive their body position in order maintain balance or walk [4]. Alterations of this ability serve as risk factors for disabilities or a worsening of health status, and can lead to reduced quality of life, particularly when falls occur [5]. Patients suffering from neurological diseases [3] or orthopedic problems [6,7] often show impaired static balance, which can also lead to gait alterations, as observed in patients with multiple sclerosis or Parkinson’s disease [8,9,10].

Static balance analyses have been well-established in the clinical environment, but often require a dedicated laboratory or expensive equipment [11]. However, the precision of such equipment (e.g., 3D motion capture systems and force plates) has been tested extensively, yielding very good reliability on the outcome measures [12,13].

An alternative to fixed systems is the application of wearable health technology, such as inertial measurement units (IMUs) [14,15]. IMUs commonly consist of accelerometers, gyroscopes, and magnetometers, and provide the opportunity to assess gait and balance directly on the clinical ward, without the need for a dedicated laboratory [16]. Such supervised assessments can be used to monitor improvements over time e.g., during hospitalization, or serve as digital clinical endpoints to quantify the success or failure of an intervention [17].

IMUs generate time series of raw data that are then digitally processed to extract spatiotemporal parameters [18,19]. Their validity has been evaluated abundantly [20], and the clinical evaluation in a large sample of neurological inpatients has been shown to be very successful [15]. Although these are promising results, assessment of day-to-day variability, reliability, and minimal detectable change (MDC) of IMU-based static balance measures is lacking.

Therefore, the aim of this study is to assess the day-to-day variability and use the results to provide hypothetical reliability and MDC measures of standardized balance assessment in a cohort of neurogeriatric patients, by using different tasks and extracting the most relevant parameters.

## 2. Materials and Methods

A convenience sample of 41 inpatients was chosen (20 men (age: 78 ± 4 years, BMI = 25.9 kg/m^2^, and 21 women (age: 79 ± 4 years, BMI = 25.1 kg/m^2^). Study participants were referred to the neurogeriatric or neurological wards of the Neurology Department at the University Hospital Schleswig-Holstein, Campus Kiel between 09/2017 and 12/2019 (the study protocol is reported in [16]).

Inclusion criteria were the presence of a neurological disorder, age of at least 60 years, ability to stand alone for at least ten seconds, and the ability to walk three meters without personal assistance (walking aids allowed) [16]. Exclusion criteria were current or past chronic substance abuse (except nicotine), corrected visual acuity < 60%, >2 falls in the previous week (risk of falling too high during the examination), ≤5 points in the Montreal Cognitive Assessment (MoCa) test [17,21], and not being able to perform at least one of the balance tasks. The ethics committee of the medical faculty of the University of Kiel approved the study (No. D427/17), and all participants gave written informed consent prior to participation. The three most common diagnoses (60% of all investigated diagnoses) were stroke (*n* = 16), Parkinson’s disease (*n* = 6), and back pain (*n* = 3).

### 2.1. Quantitative Gait and Balance Assessment

**Figure 1:**
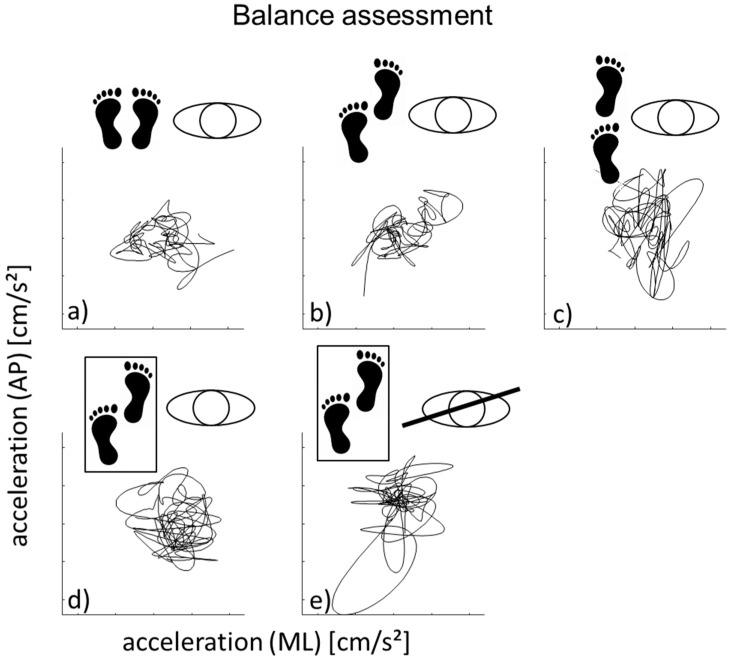
The balance assessment contains three different foot positions on a flat hard surface (side-by-side (**a**), semi-tandem (**b**), tandem (**c**)) and a condition on a balance mat (black outline) in a semi-tandem stance with eyes open (**d**) and closed (**e**). The acceleration traces in the horizontal plane (medio-lateral (M/L) and anterior-posterior (A/P) directions) for one representative participant are shown under each balance condition.

To determine the day-to-day reliability and MDC, two standardized IMU-based balance assessments were performed within 12–24 h. For each of them, participants were equipped with a wearable IMU system (Rehawatch^®^, Hasomed, Magdeburg, Germany) consisting of three IMUs worn at both ankles and at the lower back (L4–L5). Each IMU contains a 3D accelerometer (±8 g), a 3D gyroscope (±2000°/s), and a magnetometer (±1.3 Gs), resulting in nine degrees of freedom. The assessment included the following tasks: side-by-side stance, semi-tandem stance, tandem stance on hard ground with eyes open, and semi-tandem assessment on a soft surface with eyes open and closed for 30 s each (Figure 1). If the tasks on the hard surface were successfully completed, then the participants were asked to stand for 30 s on a soft pad (Airex balance pad, 50 × 41 × 6 cm) in a semi-tandem position with eyes open and eyes closed.

### 2.2. Sensor Data Processing

The IMU data was processed by custom written scripts using MATLAB (MathWorks, Nantick, MA) based on Mancini et al. [19]. The collected parameters provided information about the sway area (surface) (cm^2^/s^4^), velocity (vel) (cm/s), acceleration (acc) (cm/s^2^), jerk (cm/s^3^), and frequency (Hz). Velocity, acceleration, and jerk were expressed as the root mean square value and computed in both anterior-posterior (AP) and medio-lateral (ML) directions, as there is evidence that differences in both directions can represent different pathologies or compensation strategies of the body [19,22].

### 2.3. Statistical Analysis

The mean (M) and standard deviation (SD) values from the two measurements were calculated for the respective parameters. A paired sample t-test, was then performed to compare potential differences of the two measurements. The relative reliability (rR) was expressed by the intra-class correlation coefficient (ICC) two-way random, the average of the k raters’ measurements (2, k), and by the formula:

$$\mathrm{ICC}\;\left(2,k\right)=\frac{\mathrm{BMS}-\mathrm{EMS}}{\mathrm{BMS}+\left(\mathrm{JMS}-\mathrm{EMS}\right)/n}$$

Here, the between-target mean square (BMS), residual mean square (EMS), within participants mean square (JMS), and number of participants (n) were included in the analysis [23,24]. The ICC is used to evaluate both systematic and random errors that could affect the rR of the exercises. An ICC of >0.9 indicates excellent, >0.75–0.9 good, >0.5–0.75 moderate, and ≤0.5 poor reliability [25,26].

Absolute reliability (aR) describes the participant-internal variability that is due to repeated measurements or, in other words, describes the smallest amount of change required to designate a change as real and beyond the bounds of measurement error [26]. MDC values are important to interpret change scores, as they allow the evaluation of the effectiveness of therapeutic interventions [27].

MDC values are calculated based on the standard error of measurement (SEM). The SEM is calculated as follows:

$$\mathrm{SEM}=\mathrm{SD}\;\sqrt{1-\mathrm{ICC}}$$

The MDC, which represents the minimum detectable change in two repeated measurements that is not due to random variation in the measurements, is calculated for a 95% confidence interval as:

MDC95=SEM × 1.96x √2

1.96 is the z-value for a normally distributed two-sided table with a 95% confidence interval, and 2 is used to account for the variance of the two measurements.

The MDC95 is also expressed as a percentage, and defined as:

$$\mathrm{MDC}95\%\;=\frac{\mathrm{MDC}95}{\mathrm{mean}}\;\times \;100$$

Here, mean is the mean value of the respective parameters for all measurements of the two assessments. MDC95% represents the minimum detectable change presented as a percentage that is not due to random variations of the measurements [25].

## 3. Results

Forty-one participants completed the side-by-side stance, 39 the semi-tandem stance, and 21 the tandem stance task on a hard surface. Nineteen participants completed the semi-tandem stance on a soft surface with eyes open, and nine participants the semi-tandem stance on a soft surface with eyes closed.

### 3.1. Side-by-Side Stance

Table 1 shows that the rR was in a poor range for all parameters (ICC < 0.5). The best ICC values were reached by acc_AP_ (0.36), vel_AP_ (0.35), and frequency (0.29). The worst value was reached by jerk_ML_ (0.02). The ICC values were slightly better in the AP direction than in the ML direction. MDC95% was best for the parameters acc_ML_ = 43%, frequency = 48%, and acc_AP_ = 73%. The values were slightly better in the ML direction than in the AP direction.

### 3.2. Semi-Tandem Stance on a Hard Surface

Table 2 shows the measurement results of the semi-tandem stance on hard ground. The rRs were in a moderate range for the parameters acc_AP_ (ICC = 0.52) and surface (ICC = 0.50). All other parameters showed poor ICC values. ICC values were slightly better in the AP direction than in the ML direction. The best MDC95% values were reached by frequency (43%), acc_ML_ (51%), and acc_AP_ (54%). The worst value was reached by Jerk_ML_ (213%). The values of the parameters area, vel_AP_, vel_ML_, jerk_AP_, and jerk_ML_ were between 87–162%. Better values for acceleration and velocity were found in the ML direction than in the AP direction, and vice versa for the jerk.

### 3.3. Tandem Stance on a Hard Surface

Table 3 shows the results of the tandem stance; rR was in a poor range for all parameters (ICC < 0.5). The best values were reached by acc_AP_ (0.48) and frequency (0.35). Jerk_ML_ (ICC = 0.01) reached the worst value. ICC values were slightly better in the AP direction than in the ML direction.

The best aR was found for frequency (MDC95% = 57%) and acc_AP_ (95%). Jerk_AP_ (220%) reached the worst value. The other parameters were between 101–190%. Values for the parameters velocity and jerk were better in the ML direction than in the AP direction, and vice versa for acceleration.

### 3.4. Semi Tandem Stance a Soft Surface (Eyes Open)

Table 4 shows the measurement results of the semi-tandem stance on soft ground with open eyes. Acc_ML_ showed a good rR, with an ICC of 0.75. The parameters area, vel_AP_, vel_ML_, and acc_AP_ had a moderate ICC (0.5–0.75). The poorest rR was reached by jerk_AP_ (0.03). The best MDC95% values were reached by acc_ML_ (53%) and frequency (55%). Jerk_AP_ had the worst value (202%). The other parameters were between 77% and 200%. Better results were achieved in the ML direction than in the AP direction.

### 3.5. Semi Tandem Stance a Soft Surface (Eyes Closed)

Table 5 shows the measurement results of the semi-tandem stance on soft ground with eyes closed. The best rRs were reached by acc_ML_ (ICC = 0.65), which reflected moderate reliability. Acc_AP_, vel_ML_, surface, and frequency also reached moderate ICC values (0.50–0.60). The worst ICC was reached by jerk_ML_, jerk_AP_, and vel_AP_.

The best MDC95% values were obtained by the parameters frequency (39%) and acc_ML_ (55%). The worst value was jerk_AP_ (278%). The other values were between 66% and 197%. ML direction values were better than those in the AP direction.

### 3.6. MDC95% Values of All Parameters and Experimental Conditions

**Figure 2:**
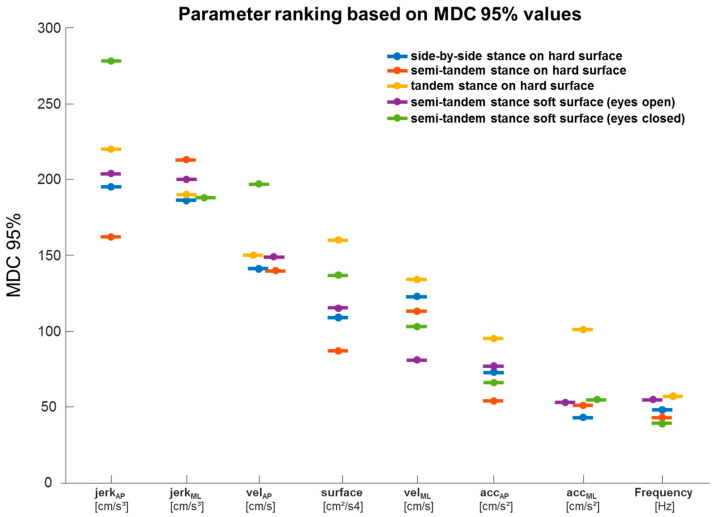
MDC95% values of all exercises and parameters ranked from high to low. *X*-axis = parameters, *Y*-axis = MDC95% values. Surface, vel_AP_ = velocity in the AP direction, vel_ML_ = velocity in the ML direction, acc_AP_ = acceleration in the AP direction, acc_ML_ = acceleration in the ML direction, jerk_AP_ = jerk in the AP direction, jerk_ML_ = jerk in the ML direction.

The MDC95% values of all exercises and associated parameters are shown in Figure 2. The parameter frequency showed good MDC95% values (39–57%) for all exercises, followed by the parameters acc_ML_ and acc_AP_, with MDC95% values of 43% to 77%. By far the worst MDC95% values were determined by the parameter jerk, with values between 162% and 278%. The parameters vel_ML_, vel_AP_, and surface were always in an MDC95% range of >100%, except for vel_ML_ in the semi-tandem stance on soft ground with open eyes, with an MDC95% = 80%, and the surface parameter in the exercise semi-tandem stance on hard ground with an MDC95% = 87%.

## 4. Discussion

In this study, IMUs were used to evaluate the day-to-day variability of static balance parameters in neurogeriatric patients. Two assessments containing five experimental conditions were performed within 24 h to evaluate the reliability using ICC [22] and MDC [28] for eight balance parameters.

The highest ICC values (between 0.5 and 0.75, which reflects moderate reliability) were obtained during the semi-tandem stance on a soft surface with eyes open and eyes closed. The highest ICC values were obtained with the parameters acc_ML_ (ICC = 0.75) and area (ICC = 0.66). Our results are in line with previous findings showing MDC95% values of 113% in older adults during single-leg stance time, which is also in a poor reliability range [29]. Our findings are in contrast with results presenting the evaluation of very coarse balance scales, such as the Berg Balance Scale in Parkinson’s disease patients, showing MDC95% values of 10–13% and ICC values of 0.87–0.95 [30,31].

When comparing cohorts using the above parameters [20,32], our results need to be interpreted with caution. The questionable reliability and high MDC values may seem surprising. However, a direct comparison with previous studies is difficult due to different cohorts and experimental protocols. The data reported by [19] contain 13 subjects with early untreated Parkinson’s disease and 12 age-matched control subjects. Their ICC values for the control group ranged between 0.60 and 0.89, and for the PD group between 0.55 and 0.86. However, the participants performed the experiment twice within 30 min, thereby reducing variability. In contrast, in our setup, measurements were repeated on the following day, thereby capturing day-to-day variability of function, which is known to be high in neurogeriatric patients. We would argue that this captures the true performance range of neurogeriatric patients more accurately than directly repeated tasks. In addition, when calculating the MDC95% using the reported ICC, SEM, and mean values from their experiment, the values also range from 14–32% for healthy controls and 20–160% for patients with Parkinson’s disease, confirming the large random variations observed in our experiments. Similarly, data from [33] contain 21 healthy subjects and 17 patients with diabetic peripheral neuropathies, and the reported ICCs were excellent (ICC(1, 1) = 0.76, F(41, 40.5) = 0.71, 95% CI = (0.57, 0.86), *p* < 10^−6^); however, the authors calculated the reliability by combining measurements with eyes open and closed from data obtained within the same measurement session.

In order to maintain static balance, two main strategies exist, the ankle and the hip strategy [34]. Depending on the difficulty of the balance task, these strategies might be used separately or together [35], especially when external perturbation below or at the feet level are introduced [36]. While the ankle strategy tries to maintain balance by only stabilizing the body about the ankle joint with minimal movement about the upper body, the hip strategy involves the upper body and influences the moment of inertia about the ankle to maintain balance [37]. The ankle strategy is expected to be employed for unperturbed stance, whereas the hip strategy is expected to be employed for perturbations, or when the support surface is soft and little ankle torque can be applied [38]. Consequently, the results for the simple exercises (side-by-side stance and semi-tandem stance on hard ground) were better (therefore more reliable) on average, and for the more difficult exercises (semi-tandem stance on soft ground with open/closed eyes and tandem stance) were worse (less reliable), which could also be related to the choice of strategy.

As a consequence, even though IMUs have already been proven to be a reliable instrument during the timed up and go test [39], walking [40], and a way to measure static balance under clinical and out of hospital conditions [16,19,20,41,42], not every experimental condition and extracted parameter seem equally suitable for clinically relevant measurements [43].

There are limitations of this study that may explain the low ICCs and large MDC values. Firstly, there may be some learning effect from the first to the second assessment. However, we do not feel that this substantially influenced the results, as we did not see in the plots a systematic improvement of parameters during the second assessment compared to the first. Secondly, different performance between day one and day two has already been described in a sit-to-stand test [25] and balance and ambulation tests [31]. This is exactly what we want to measure in studies as presented here, and it may be that patients with neurogeriatric conditions may be particularly prone to fluctuations of performance between two days. In fact, we know especially from Parkinson’s disease that long-term fluctuations can be regularly observed [44]. Finally, group sizes were different across the conditions investigated. This may have additionally influenced the outcome parameters, such as the ICC and the MDC95% [45,46], as larger group sizes could potentially increase the reliability.

## 5. Conclusions

Our results support previous studies reporting that static balance can be assessed with IMUs. However, reliability of the extracted parameters remains questionable in a neurogeriatric cohort. The most stable parameters were acceleration (especially in the ML direction) and frequency, which may thus have the highest potential to reflect disease progression and response to treatment. Future reliability studies are urgently needed investigating additional neurogeriatric cohorts, healthy control groups, and other diseased cohorts to fully understand the potential of mobile health technology-derived parameters that are generally considered as highly accurate.

## Figures and Tables

**Table 1:** Reliability and minimal detectable change: side-by-side stance on a hard surface (*n* = 41).

Parameter	Mean_t1_	SD_t1_	Mean_t2_	SD_t2_	*p*_t1-T2_	ICC	MDC95%
SURFACE (CM^2^/S^4^)	20.9	9.8	20.1	9.1	0.657	0.26	109
VEL_AP_ (CM/S)	2.62	1.51	2.34	1.62	0.336	0.35	141
VEL_ML_ (CM/S)	1.02	0.45	0.88	0.43	0.160	0.11	123
ACC_AP_ (CM/S^2^)	1.24	0.43	1.19	0.37	0.469	0.36	73
ACC_ML_ (CM/S^2^)	0.87	0.16	0.84	0.14	0.413	0.21	43
JERK_AP_ (CM/S^3^)	1072	832	1110	765	0.827	0.06	195
JERK_ML_ (CM/S^3^)	944	765	987	534	0.777	0.02	186
FREQUENCY (HZ)	1.57	0.34	1.55	0.30	0.735	0.29	48

**Table 2:** Reliability and minimal detectable change: semi-tandem stance a hard surface (*n* = 39).

Parameter	Mean_t1_	SD_t1_	Mean_t2_	SD_t2_	*p*_t1-T2_	ICC	MDC95%
SURFACE (CM^2^/S^4^)	23.5	10.8	22.4	9.5	0.538	0.5	87
VEL_AP_ (CM/S)	2.29	1.30	2.27	1.42	0.950	0.28	140
VEL_ML_ (CM/S)	1.26	0.64	1.27	0.57	0.946	0.27	113
ACC_AP_ (CM/S)	1.19	0.35	1.19	0.33	0.937	0.52	54
ACC_ML_ (CM/S^2^)	1.09	0.32	1.02	0.19	0.122	0.45	51
JERK_AP_ (CM/S^3^)	1067	586	974	641	0.507	0.06	162
JERK_ML_ (CM/S^3^)	1057	833	976	711	0.660	0.03	213
FREQUENCY (HZ)	1.70	0.32	1.62	0.32	0.151	0.34	43

**Table 3:** Reliability and minimal detectable change: tandem stance on a hard surface (*n* = 21).

Parameter	Mean_t1_	SD_t1_	Mean_t2_	SD_t2_	*p*_t1-T2_	ICC	MDC95%
SURFACE (CM^2^/S^4^)	43.2	25.6	46.4	34.5	0.736	0.25	160
VEL_AP_ (CM/S)	3.62	2.00	3.22	2.35	0.500	0.27	150
VEL_ML_ (CM/S)	1.97	0.99	1.71	0.96	0.377	0.16	134
ACC_AP_ (CM/S^2^)	1.48	0.60	1.69	0.88	0.238	0.48	95
ACC_ML_ (CM/S^2^)	1.83	0.71	1.65	0.78	0.397	0.26	101
JERK_AP_ (CM/S^3^)	1632	1211	964	889	0.044	0.13	220
JERK_ML_ (CM/S^3^)	1265	785	1230	953	0.901	0.01	190
FREQUENCY (HZ)	1.72	0.46	1.69	0.42	0.812	0.35	57

**Table 4:** Reliability and minimal detectable change: semi-tandem stance a soft surface (eyes open) (*n* = 19).

Parameter	Mean_t1_	SD_t1_	Mean_t2_	SD_t2_	*p*_t1-T2_	ICC	MDC95%
SURFACE (CM^2^/S^4^)	49.3	35.3	49.2	35.5	0.992	0.66	115
VEL_AP_ (CM/S)	8.44	6.06	9.05	7.71	0.703	0.53	149
VEL_ML_ (CM/S)	3.23	1.63	2.88	1.41	0.296	0.65	81
ACC_AP_ (CM/S^2^)	1.76	0.79	1.82	0.72	0.704	0.55	77
ACC_ML_ (CM/S^2^)	1.41	0.51	1.36	0.56	0.564	0.75	53
JERK_AP_ (CM/S^3^)	1339	1035	1363	1009	0.941	0.03	204
JERK_ML_ (CM/S^3^)	1207	1018	1119	739	0.753	0.09	200
FREQUENCY (HZ)	1.57	0.48	1.58	0.41	0.925	0.48	55

**Table 5:** Reliability and minimal detectable change: semi-tandem stance soft ground with closed eyes (*n* = 9).

Parameter	Mean_t1_	SD_t1_	Mean_t2_	SD_t2_	*p*_t1-T2_	ICC	MDC95%
SURFACE (CM^2^/S^4^)	105.3	74.9	71.9	43.0	0.125	0.5	137
VEL_AP_ (CM/S)	9.67	7.06	8.56	6.75	0.719	0.08	197
VEL_ML_ (CM/S)	6.14	3.85	4.75	2.19	0.139	0.59	103
ACC_AP_ (CM/S^2^)	2.55	1.00	2.01	0.62	0.025	0.6	66
ACC_ML_ (CM/S^2^)	2.14	0.71	1.93	0.76	0.302	0.69	55
JERK_AP_ (CM/S^3^)	1067	897	1055	942	0.98	−0.41	278
JERK_ML_ (CM/S^3^)	1171	573	1025	834	0.678	−0.13	188
FREQUENCY (HZ)	1.58	0.30	1.69	0.39	0.364	0.54	39
